# The influence of (central) auditory processing disorder on the severity of speech-sound disorders in children

**DOI:** 10.6061/clinics/2016(02)02

**Published:** 2016-02

**Authors:** Nadia Vilela, Tatiane Faria Barrozo, Luciana de Oliveira Pagan-Neves, Seisse Gabriela Gandolfi Sanches, Haydée Fiszbein Wertzner, Renata Mota Mamede Carvallo

**Affiliations:** Faculdade de Medicina da Universidade de São Paulo, Departamento de Fisoterapia, Ciência da Comunicação & Transtornos, Terapia Ocupacional, São Paulo/, SP, Brazil

**Keywords:** Phonological Impairments, Speech Articulation Tests, Auditory Perceptual Disorders, Child, Auditory Perception

## Abstract

**OBJECTIVE:**

To identify a cutoff value based on the Percentage of Consonants Correct-Revised index that could indicate the likelihood of a child with a speech-sound disorder also having a (central) auditory processing disorder**.**

**METHODS:**

Language, audiological and (central) auditory processing evaluations were administered. The participants were 27 subjects with speech-sound disorders aged 7 to 10 years and 11 months who were divided into two different groups according to their (central) auditory processing evaluation results.

**RESULTS:**

When a (central) auditory processing disorder was present in association with a speech disorder, the children tended to have lower scores on phonological assessments. A greater severity of speech disorder was related to a greater probability of the child having a (central) auditory processing disorder. The use of a cutoff value for the Percentage of Consonants Correct-Revised index successfully distinguished between children with and without a (central) auditory processing disorder.

**CONCLUSIONS:**

: The severity of speech-sound disorder in children was influenced by the presence of (central) auditory processing disorder. The attempt to identify a cutoff value based on a severity index was successful.

## INTRODUCTION

The heterogeneity of children with speech-sound disorders (SSDs) suggests that disorder subtypes should be defined to establish effective tests and specific diagnostic criteria for more accurate assessments of speech-sound errors.

The Percentage of Consonants Correct-Revised (PCC-R) index 1,2 is one of the most commonly applied indexes used to quantify the severity of speech impairment in children with SSDs during both evaluation and intervention 1,2,3,4,5. This quantitative measure is highly sensitive to differences in phonological deficits because it provides information pertaining to the two main error types: omissions and substitutions 1,2.

A child with a SSD may have an input problem characterized by temporary hearing loss, which is usually caused by fluctuating conductive hearing loss associated with early recurrent otitis media with effusion, a (central) auditory processing disorder ((C)APD) (affecting the perception of sounds), a problem with speech production output (e.g., dysarthria or mislearning of the motor program in articulating specific speech sounds), or even a cognitive-linguistic deficit that manifests as difficulty in phonological processing at the phonemic level 6,7. Several researchers 7,8,9 have studied these three areas separately using different methods, which makes it difficult to identify one or more of them as the basis for the difficulties observed in children with SSDs. Studies on speech motor system development have emphasized that until approximately their second birthday, children are still building a phonological system based on listening (auditory feedback) 10 and sensory experiences, allowing them to produce specific sounds as soon as they develop the motor control necessary to perform the movements required for speech. This auditory feedback is crucial for phonological development and the integrity of (central) auditory processing plays an important role in this process, which culminates in the auditory perception of sound.

A (C)APD may cause certain speech disorders because it interferes with the formation of a stable representation of phonemes in the brain and with speech perception, making the learning of phonology, syntax and semantics difficult 11. A failure of or interference in the cortical processing of auditory information is expected to affect the integration, understanding and, ultimately, interpretation of sound stimuli 12.

Auditory perceptual difficulties related to (central) auditory processing have been studied in children with SSDs 11,, but few investigations have been conducted to evaluate the influence of (C)APDs on SSDs 16. In addition, little is known regarding the relationship between (C)APDs and SSD severity in children.

Recent studies have sought to identify such a relationship. A study of (central) auditory processing performance in 44 subjects revealed that children with SSDs underperformed on tests of (central) auditory processing compared with the typically developing group 13. Muniz et al. 17 verified that children with SSDs may exhibit a temporal processing disorder. Caumo and Ferreira 18 demonstrated that children who exhibited other phonological processes that interfered with syllable structure (in addition to sound substitutions) had more difficulty with auditory processing tests than those who produced only sound substitutions. A study 14 on the severity of SSDs in children with and without a (C)APD indicated that the group without a (C)APD tended to present higher PCC-R values, indicating less severe phonological impairment 2. The identification of certain patterns during phonological assessments, from which auditory perceptual difficulties can be inferred, can help in modeling and selecting the priorities for speech intervention.

Based on the studies listed above, this research was motivated by the fact that the effects of (C)APDs in this population are not well elucidated. This lack of information is possibly attributable to the age at diagnosis of the speech difficulty being between ages 4 and 6 years, whereas (C)APD evaluation is conducted mainly after the age of 7 years. Our hypothesis was that children with SSDs and (C)APDs have a more severe degree of speech impairment than children with SSDs and no (C)APD. Our main objectives were the following: To compare the PCC-R between SSD children with and without a (C)APD;

To identify a cutoff value based on the PCC-R index that could indicate the likelihood of a child with an SSD also having a (C)APD;

To establish sensitivity and specificity values for the PCC-R index in identifying children with a (C)APD associated with a SSD.

## METHODS

### Ethics Statement

All the subjects or their parent/guardian (for minors) provided written informed consent to participate in the study, which was performed with approval of the Institutional Review Board of the Faculty of Medicine at University of São Paulo (protocol number 201/11).

### Subjects

The participants were 27 subjects with SSDs, aged 7 to 10 years and 11 months, who were divided into two different groups according to their (central) auditory processing evaluation results.

The inclusion criteria were as follows: diagnosis of a SSD based on the picture-naming and word imitation tasks from the phonology test 19 and age-expected responses for the vocabulary, pragmatics and fluency tests, all of which are derived from the Infantile Language Test-ABFW 20. In addition, inclusion required confirmation of hearing thresholds below 20 dB HL at 500, 1000, 2000 and 4000 Hz, as well as normal tympanometry and acoustic reflexes. The children were monolingual Brazilian-Portuguese speakers and could not have been enrolled in any type of speech and language therapy previously or at the time of the evaluation process. Children with associated deficits in the vocabulary, pragmatics and/or fluency tests were excluded from the study.

To be diagnosed with a SSD, the child had to have a delay in the development of or disorder of the phonological system with no impairment in other language areas (vocabulary, pragmatics and fluency) in the absence of neurological damage, sensorineural hearing loss, or facial malformation. Children presenting only misarticulations (e.g., sound distortions) were not included.

After specific language tests, the subjects were given an audiological evaluation.

Based on the (central) auditory processing evaluation, the children were divided into two groups: Group 1 (G1) included 13 subjects with SSDs and without (C)APDs (aged 7.2 to 10.7 years, mean age of 8.8, SD=1.0) and Group 2 (G2) included 14 subjects with SSDs and (C)APDs (aged 7.1 to 8.10 years, mean age of 8.1, SD=0.7).

### Phonological Evaluation

The phonology test 19, derived from the ABFW Infantile Language Test, which was developed and standardized for Brazilian Portuguese native-speakers, was used. The test includes an evaluation of four language domains: vocabulary, pragmatics, phonology and fluency. The phonological evaluation consists of a picture-naming task with 34 pictures (90 consonants) and an imitation-of-words task with 39 words (107 consonants) 19. The children's performance was audio- and video-recorded. The percentage of consonants correct-revised (PCC-R) index 1,2 was calculated separately for the picture-naming task (PCC-RN) and the imitation-of-words task (PCC-RI) by dividing the number of correct consonant sounds by the total number of consonants in the sample and multiplying by 100. This quantitative measure was chosen because it is highly sensitive to differences in phonological deficits, providing information pertaining to the two main error types: omissions and substitutions (distortions are not accounted for).

### Audiological Evaluation

The children underwent tympanometry and ipsilateral acoustic reflex threshold assessments (middle ear analyzer: Interacoustics® AT235 h, Middelfart, Assens, Denmark). Pure-tone audiometry (air conduction) and a speech test (SRT–speech recognition threshold) were conducted using a GSI 61 audiometer GrasonStadler® (ANSI S3,6-1989; ANSI S3,43-1992; IEC 645-1,1992; IEC 645 – 2, 1993; ISO 389; UL 544) with testing frequencies ranging from 0.25 to 8 kHz, in octave intervals (headphones TDH 50P).

### (Central) Auditory Processing Evaluation

Three different auditory skills were tested by the Brazilian versions of four (central) auditory processing tests 21. Auditory closure was assessed using the Figure Identification (with competitive ipsilateral noise) (FI) Test; binaural integration was assessed using the Dichotic Digits (DD) Test; and temporal ordering was tested using both the Pitch Pattern Sequence (PPS) Test and the Duration Pattern Sequence (DPS) Test 22. The FI Test is composed of 10 words presented with ipsilateral competing white noise (signal-to-noise ratio +20 dB because the noise was effective). The speech stimuli were presented at 40 dB SL (with reference to the SRT). The child was asked to point to the figure whose name was heard. The DD Test (Brazilian Portuguese version) is composed of 20 pairs of two digits that are presented simultaneously in each ear (dichotic paradigm) at 50 dB SL (with reference to the SRT). The child was asked to repeat the digits and a percentage correct score was calculated for each ear. The PPS Test (pediatric version) is composed of 30 sequences, each comprised of three tones. Each sequence consists of a combination of a low-frequency tone (L=880 Hz) and a high-frequency tone (H=1430 Hz), with a total tone duration of 500 ms (10 ms rise-fall time) and an inter-stimulus interval of 300 ms. In each sequence, two of the three tones had the same frequency. Six different sequences of three tones were generated and presented randomly at 50 dB SL (with reference to the SRT). The child was asked to verbally describe each sequence heard. The DPS Test is similar to the PPS Test and involves the presentation of 30 sequences comprised of three tones. However, the frequency of the tones was maintained at 1000 Hz and the duration of the tones was either short (S=250 ms) or long (L=500 ms). In each sequence, two of the three tones had the same duration. A total of six different patterns were randomly presented at 50 dB SL (with reference to the SRT). The children were also asked to verbally describe each sequence they heard. The PPS and DPS tests were selected to evaluate temporal processing in children with SSDs because temporal frequency and duration patterns are very important in the development of the phonological system in these children. The test results were classified as normal or impaired according to the normative standardized patterns for Brazilian Portuguese speakers 21,23.

According to the (central) auditory processing test results, the children were classified as normal when the results of at least three of the four tests were in the normal range. The children were classified as having (C)APD when the results of two or more tests were below the scores established by normative data 24.

### Statistics

Student's t-test was used to compare the mean age between G1 and G2. The PCC-R index was compared between groups using the Mann-Whitney test. For this comparison, the Mann-Whitney test was used because the results did not meet the requirements (e.g., having a normal distribution) for the application of parametric tests.

Receiver operator characteristic (ROC) curves were created by plotting the sensitivity *vs*. specificity values for the PCC-RI and PCC-RN of all the subjects in an attempt to determine the need to administer a (central) auditory processing evaluation to a child with an SSD. The rules of this analysis were established to determine a cutoff value for the dependent variables that provided the highest sensitivity and specificity for each case. In the present study, sensitivity was defined as the percentage of children who had a PCC-R score below the cutoff value and also presented with a (C)APD. In addition, specificity was defined as the percentage of children who had a PCC-R score above the cutoff value and had normal (central) auditory processing evaluation results.

The number of auditory skills (auditory closure, binaural integration, temporal ordering) considered to be impaired according to the normal-range values was calculated as 0 (no auditory skill scores below normal), 1, 2, or 3 (all auditory skill scores below the normal range). The Kruskal-Wallis test was used to verify the association between the PCC-R score and the number of impaired skills. The PCC-R distribution was calculated according to the number of impaired skills (0, 1, 2, or 3). A Bonferroni correction was used when necessary.

The PCC-R distributions were compared using the Mann-Whitney test between G1 and G2 for each of the three auditory skills tested. Fisher's exact test was applied to test the hypothesis of the lack of an association between the PCC-R score and auditory skills.

The significance level was considered to be 0.05. The software program used for the statistical analysis was the SPSS predictive analytics software (version 18).

## RESULTS

### Sample Characterization

There was no significant difference in age between the groups (*p*=0.063). The results from the (central) auditory processing tests indicated that five children in G1 had only one test score that indicated impairment (Table 1).

### Comparison of the mean PCC-R scores

A comparison of the mean PCC-R scores across the three auditory skills tested in the (central) auditory processing evaluation is presented in Table 2 according to the number of subjects classified as above and below the normal range for each auditory skill. The results indicated a difference in both of the PCC-R indexes (PCC-RI and PCC-RN) between the subjects with scores classified as above and below the normal ranges only for the binaural integration skill.

### Distribution of the subjects according to the number of impaired auditory skills

The results of the comparison between the PCC-RN and PCC-RI values and the four impaired skills (0, 1, 2, or 3) indicated that the distributions of the PCC-RI values (*p*=0.028) and PCC-RN values (*p*=0.037) were not equal. Based on this finding, a pairwise comparison of the PCC-R values using the Bonferroni correction method according to the number of impaired skills was applied. Evidence of a significant difference in the distribution of PCC-R values for both phonological tasks (picture naming and imitation of words) was observed between the children who had just one impaired auditory skill and the children with impairment of all three auditory skills (*p*=0.023 PCC-RI; *p*=0.036 PCC-RN).

**Figure 1:**
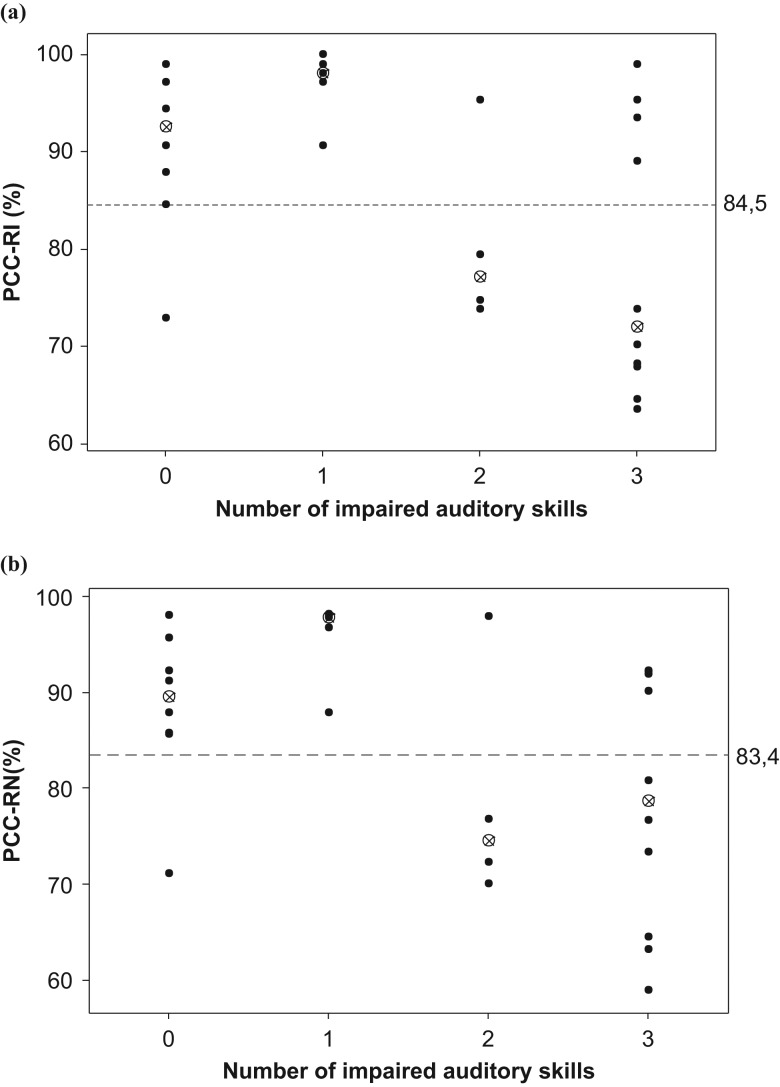
Distribution of the children according to the number of impaired auditory skills associated with Percentage of Consonants Correct-Revised I (a) and Percentage of Consonants Correct-Revised N (b) values.

The distribution of the subjects according to the number of impaired auditory skills (0, 1, 2, or 3) based on the PCC-R value is presented in Figure 1 for the imitation-of-words task (a) and the picture-naming task (b).

### Cutoff value determination

**Figure 2:**
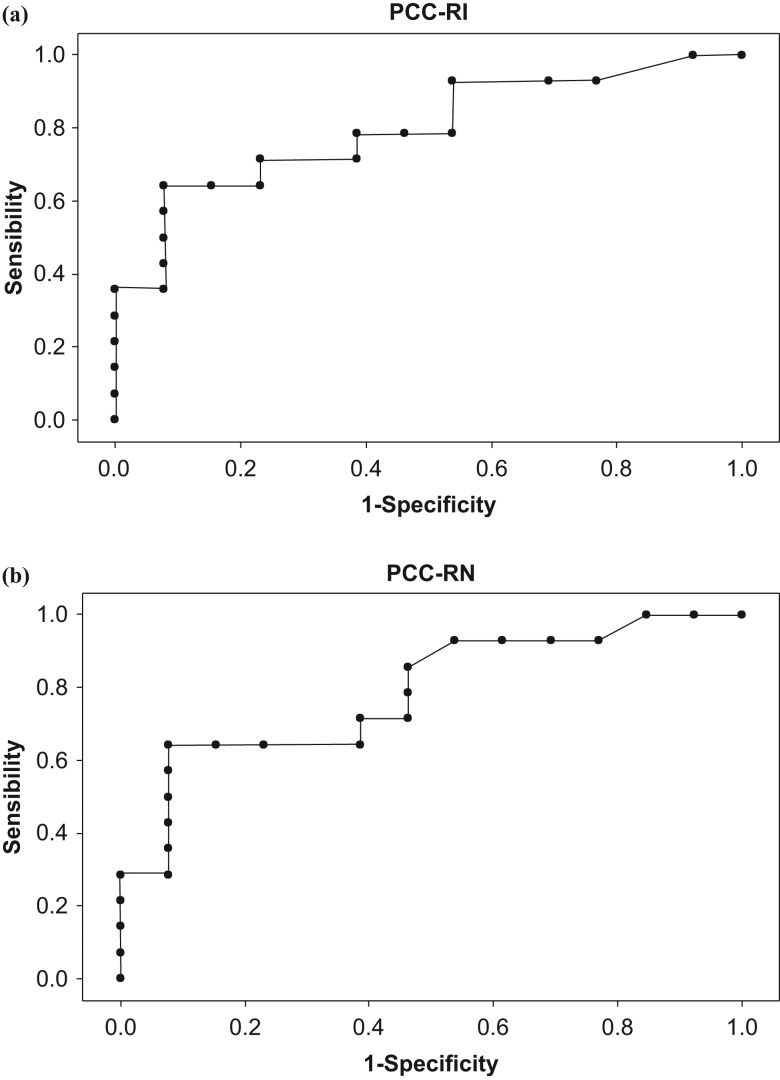
ROC curves for the Percentage of Consonants Correct-Revised I (a) and the Percentage of Consonants Correct-Revised N (b) values.

The ROC curves (Figure 2) were generated in an attempt to determine the need to administer a (central) auditory processing evaluation to a child with an SSD. The cutoff value for the PCC-RI score was 84.5%, which corresponds to a sensitivity of 0.64 and specificity of 0.92. The cutoff value for the PCC-RN score was 83.4% (sensitivity of 0.64 and specificity of 0.92). These findings indicate that below the two cutoff values (for the picture-naming and imitation-of-words tasks), the child may have a (C)APD associated with the SSD. The area under the curve values indicate good discriminatory power of the PCC-R score to identify an individual as having or not having a (C)APD. Five individuals in G2 (35.7%) presented values above the cutoff value, which were considered as false-negatives. In G1, only one subject (7.7%) had a score below the cutoff value, which was considered to be a false-positive.

The frequency distribution and percentage scores for the three different skills tested in the (central) auditory processing evaluation are presented according to the cutoff value of 80%, as established by the ROC curves for the PCC-R index in Table 3. The results are independent of the type of phonology test used (picture-naming and imitation-of-words tasks) because exactly the same individuals were below (or above) the cutoff values for the PCC-RN and PCC-RI. There was an association between the PCC-R index and binaural integration (*p*=0.001) and a tendency toward significance for the association between the PCC-R index and temporal ordering (*p*=0.097). There was no association between the PCC-R index and auditory closure (*p*=0.230).

### Distribution of the subjects according to the cutoff values

**Figure 3:**
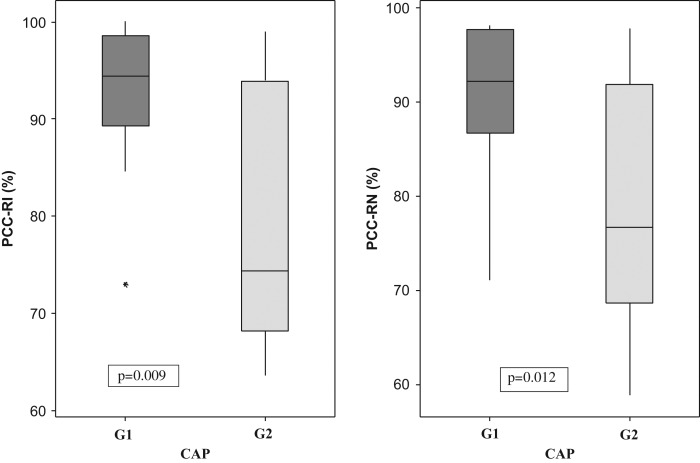
Box-plot graph of the distribution of the PCC-R index calculated for both the Percentage of Consonants Correct-Revised N and Percentage of Consonants Correct-Revised tasks for G1 and G2.

The box-plot graph (Figure 3) shows the distribution of the PCC-R indexes calculated for both the picture-naming (PCC-RN) and imitation-of-words (PCC-RI) tasks for G1 and G2. The differences in the PCC-R distribution indicate that there was greater variability among the children in G2 (children with (C)APDs). Additionally, the majority of children in G1 (without (C)APDs) presented PCC-R values above 80% on both phonology tasks. There was a significant difference between the groups in both the PCC-RI (*p*=0.009) and PCC-RN (*p*=0.012) indexes.

## DISCUSSION

The main motivation for the present study was based on the fact that if a child still has a SSD after 7 years of age, he or she may experience specific difficulties that preclude the acquisition of certain speech sounds. A possible explanation for this finding may be a (C)APD. Our initial hypothesis, i.e., that children with both a SSD and (C)APD have more severe phonological difficulties than children with a SSD and no (C)APD, was confirmed. The analysis also indicated a cutoff value for the disorder severity at which children are more likely to have a (C)APD.

The relationship between auditory perception, cognitive-linguistic processing, and motor processing has been described by different authors. The DIVA model 25,26,27 provides a well-defined framework for guiding the interpretation of experimental results related to the putative human speech mirror system and emphasizes the importance of combining the information from these processes for speech development. Other authors 28 have shown that the relationship between the acquisition of the acoustic characteristics of language and auditory and motor representations is symbiotic, i.e., one depends on the other for construction and maturation to be effective.

The results of our study also indicated that binaural integration (measured using the DD Test) is the skill that is most strongly associated with disorder severity. Dichotic tests have been used to identify lesions of the left temporal lobe. Abnormal DD Test results have been reported in both ears in patients with lesions of the left temporal lobe 29. Individuals with cortical lesions outside the temporal lobe do not exhibit inferior performance on the DD Test compared with control groups 30. Studies on patients with temporal lobe epilepsy have also indicated a worse performance on the DD Test in both ears compared with control groups 31,32. These studies demonstrate the importance of the temporal lobe in sound processing during a dichotic task. The posterior superior temporal lobe (predominantly the left side) 33 plays an important role during the various stages in the development of both speech perception and speech production. Mild cortical dysfunctions in this area may explain both the phonological difficulties and deficits observed in the DD Test. Although the relationship between perception and speech appears to be clear, it is still a challenge to determine whether difficulties in auditory processing and speech are consequences of a base deficit (cortical) or whether one process influences the other (cause and effect). The association in the present study between SSD severity and worse linguistic sound processing performance in a dichotic task strongly indicates temporal lobe deficiencies in these children.

The mean PCC-RN and PCC-RI scores also varied according to the number of impaired auditory skills, demonstrating that children who have one impaired skill have less severe deficits than children with three impaired auditory skills. These results not only strengthen the relationship between SSD severity and the presence of (C)APDs in these children but also suggest that, in children with SSDs, it is important to perform a (central) auditory processing evaluation.

The average PCC-R value measured from the speech samples in both the picture-naming and imitation-of-words tasks was higher in the group of children without (C)APD (G1), indicating that they had less severe phonemic impairments. We observed that children in this group presented less variability in this measurement compared with subjects with a (C)APD. Despite the heterogeneity between the children with a SSD in relation to speech disorder severity, this result suggests that when these children have a (C)APD in addition to speech-language disorders, they tend to be more severely affected 13,14. The results also indicate that a greater SSD severity indicates a greater likelihood of having a (C)APD.

One of the major aims of the present study was to identify a cutoff value for the PCC-R index that could be used as a severity measure in children with SSD to identify those with a higher probability of having a (C)APD. Our attempt to correlate the severity of SSD with the possibility of (central) auditory processing difficulties may be very helpful to speech-language pathologists in their decision-making regarding diagnostic and therapeutic interventions.

The cutoff values, sensitivities and specificities of the PCC-RN and PCC-RI indexes were similar; therefore, the same subjects were classified as normal or having impaired (central) auditory processing using both phonology tasks. The analysis showed that the PCC-R index had higher specificity, which indicates that this test is effective in identifying true-negatives (i.e., subjects who were considered likely to have a normal (central) auditory processing capacity). This study indicated that children presenting a PCC-R score below 84.5% in the imitation-of-words task and below 83.4% in the picture-naming task tended to have a (C)APD. It appears that there is a very high likelihood of children with PCC-R scores below the cutoff value having a (C)APD.

The moderate sensitivity (64%) of this assessment suggests that children who had PCC-R scores above the cutoff values are not exempt from having an auditory perceptual difficulty. There are children with (C)APDs who do not have speech disorders because these children, despite having a (C)APD, are able to establish neural pathways to overcome auditory perceptual difficulties and ensure proper speech development. Thus, we must also be aware of possible auditory processing disorders in children who score above the cutoff value. However, our finding indicates that a child who scores below this cutoff value is highly likely to also exhibit a (C)APD.

The combination of the results from the (central) auditory processing evaluation and the phonological assessments provides a basis for developing a more suitable intervention 34. The effectiveness of the treatment is directly related to the specificity of the diagnosis 35. Additional therapeutic strategies emphasizing the development of (central) auditory processing skills may be very helpful in treating children with SSDs who present a severity index worse than the cutoff value of 83.4% for the picture-naming task (PCC-RN) and 84.5% for the imitation-of-words task (PCC-RI). This finding is especially important for clinicians who may consider two different possible speech and language interventions for children with SSDs as follows: 1) if the child is younger than 7 years and presents a PCC-R value under 83.4% for the picture-naming task and 84.5% for the imitation-of-words task, the clinician should be encouraged to strengthen auditory skills during the therapeutic intervention; 2) if the child is older than 7 years and presents a PCC-R value under the cutoff value, the clinician should refer the child for a (central) auditory processing evaluation to determine which auditory skill(s) are most affected.

It is noteworthy that the PCC-R values established for the children in the present study can be used to facilitate clinical observation and prioritization of (C)AP evaluations. However, even if a child has higher values than the cutoff values, it is important that the therapist is aware of other features that may indicate a possible (C)APD and request an evaluation if necessary.

The findings of the present study may have practical implications. The severity of SSDs in children was influenced by the presence of (C)APDs, emphasizing the importance of using different strategies to stimulate auditory skills during therapeutic interventions in children with SSDs. Despite the small number of subjects, our attempt to identify a cutoff value based on a severity index (PCC-R) was successful, as the cutoff enables speech-language pathologists to establish more specific guidelines for the treatment and, consequently, prognosis of children with SSDs.

## AUTHOR CONTRIBUTIONS

Carvalho RM, Vilela N and Wertzner HF conceived and designed the experiments. Vilela N and Barrozo TF performed the experiments. Vilela N, Barrozo TF, Pagan-Neves LO, Sanches SG, Wertzner HF and Carvalho RM analyzed the data. Vilela N, Barrozo TF, Pagan-Neves LO and Sanches SG wrote the manuscript. Wertzner HF and Carvalho RM revised the manuscript.

## Figures and Tables

**Table 1:** The number of children who scored below the normal range for the tests.

	**FI**	**DD**	**PPS**	**DPS**
G1 (n=13)	4	0	0	1
G2 (n=14)	13*	13*	9*	8*

FI = Figure Identification in Noise Test; DD = Dichotic Digits Test; PPS = Pitch Pattern Sequence Test; DPS = Duration Pattern Sequence Test.

**<?ENTCHAR ast?>:** G2 scored below the normal range on 2 or more tests.

**Table 2:** Comparison of the number of PCC-RI and PCC-RN scores above and below the normal range for each skill.

	**Auditory Closure**	**N**	**Mean**	*p***-value**	**Binaural integration**	**N**	**Mean**	*p***-value**	**Temporal Ordering**	**N**	**Mean**	*p***-value**
PCC-RI	Above ^(a)^	10	89.9	0.315	Above ^(a)^	14	92.9	0.004*	Above ^(a)^	13	90.8	0.106
		Below ^(b)^	17	83.2		Below ^(b)^	13	77.9		Below ^(b)^	13		81.4
		Total	27	85.7		Total	27	85.7		Total	26^(c)^	86.1	
PCC-RN	Above ^(a)^	10	88.1	0.379	Above ^(a)^	14	91.6	0.002*	Above ^(a)^	13	89.2	0.166
		Below ^(b)^	17	82.6		Below ^(b)^	13	77.1		Below ^(b)^	13		81.2
		Total	27	84.6		Total	27	84.6		Total	26^(c)^	85.2	

Mann-Whitney test

**Table 3:** Association between the cutoff value for the Percentage of Consonants Correct-Revised and the auditory skills tested during the (central) auditory processing evaluation.

	Auditory Closure				Binaural Integration				Temporal Ordering			
PCC-R	Normal	Impaired	Total	*p*-value	Normal	Impaired	Total	*p*-value	Normal	Impaired	Total	*p*-value
Above^(a)^	2	8	10	0.23	1	9	10	0.001*	2	7	9	0.097
	20.0%	80.0%	100.0%			10.0%	90.0%		100.0%		22.2%		77.8%	100.0%
Below^(b)^	8	9	17			13	4		17		11		6	17
	47.1%	52.9%	100.0%			76.5%	23.5%		100.0%		64.7%		35.3%	100.0%
Total	10	17	27		14	13	27		13	13	26	
	37.0%	63.0%	100.0%		51.9%	48.1%	100.0%		50.0%	50.0%	100.0%	

Fisher's exact test.
